# High Urinary Tungsten Concentration Is Associated with Stroke in the National Health and Nutrition Examination Survey 1999–2010

**DOI:** 10.1371/journal.pone.0077546

**Published:** 2013-11-11

**Authors:** Jessica Tyrrell, Tamara S. Galloway, Ghada Abo-Zaid, David Melzer, Michael H. Depledge, Nicholas J. Osborne

**Affiliations:** 1 European Centre for Environment and Human Health, University of Exeter Medical School, Truro, United Kingdom; 2 College of Life and Environmental Sciences, University of Exeter, Exeter, United Kingdom; 3 Epidemiology and Public Health, University of Exeter Medical School, Exeter, United Kingdom; University of Cincinnati, United States of America

## Abstract

**Background:**

In recent years there has been an exponential increase in tungsten demand, potentially increasing human exposure to the metal. Currently, the toxicology of tungsten is poorly understood, but mounting evidence suggests that both the elemental metal and its alloys have cytotoxic effects. Here, we investigate the association between tungsten and cardiovascular disease (CVD) or stroke using six waves of the National Health and Nutrition Examination Survey (NHANES).

**Methods:**

We investigated associations using crude and adjusted logistic regression models in a cohort of 8614 adults (18–74 years) with 193 reported stroke diagnoses and 428 reported diagnoses of CVD. We also stratified our data to characterize associations in a subset of younger individuals (18–50 years).

**Results:**

Elevated tungsten concentrations were strongly associated with an increase in the prevalence of stroke, independent of typical risk factors (Odds Ratio (OR): 1.66, 95% Confidence Interval (95% CI): 1.17, 2.34). The association between tungsten and stroke in the young age category was still evident (OR: 2.17, 95% CI: 1.33, 3.53).

**Conclusion:**

This study represents the most comprehensive analysis of the human health effects of tungsten to date. Individuals with higher urinary tungsten concentrations have double the odds of reported stroke. We hypothesize that the pathological pathway resulting from tungsten exposure may involve oxidative stress.

## Introduction

Tungsten (also known as wolfram, W) is a strong, flexible transition metal, with a high melting point. These traits have led to it becoming an indispensable part of a broad range of industrial and commercial processes and devices that are key in modern lifestyles [1]. Tungsten metal and its alloys occur in consumer products such as electronics, light bulb filaments, cemented tungsten carbide grinding wheels, carbide tipped tools and armaments [1].

Low level human exposure to tungsten occurs from the atmosphere, drinking water and diet. Entry into the air and water occurs during tungsten ore processing, alloy fabrication, tungsten carbide production and use, as well as during municipal waste combustion [2]. Currently, human exposures from air, drinking water, and food are thought to be very low. The concentration of tungsten in ambient air is approximately <10 ng/m^3^ [3]. However, occupational exposure may result in higher body burdens.

Recent reports have suggested that tungsten should be considered as an emerging chemical toxicant of concern, [2] as its chemical stability in the environment has been questioned. When soil pH falls, tungsten becomes increasingly soluble and can leach into underlying aquifers [3]. With production of tungsten steadily rising (72,000 tons produced last year compared to 40,000 tons in 2002 [4]), and use becoming more widespread, the potential for tungsten to further contaminate the environment is increasing. Tungsten is known to be capable of biological interaction and disruption of biochemical pathways and therefore the human health impact must be considered [5].

Current knowledge of the toxicology of tungsten is fragmentary [6]. To date no significant human health effects have been attributed solely to tungsten exposure, although *in vitro* and animal studies point to tungsten toxicity leading to pulmonary inflammation [7] and the development of cancer [8]. Interestingly, a recent epidemiological study suggests that tungsten exposure may be linked to an increased risk of composite cardiovascular and cerebrovascular disease (CCVD) [9]. There is also some evidence of toxicity of “hard metal”, which is an alloy or encapsulated mixture, primarily composed of tungsten or tungsten carbide and cobalt. Other tungsten alloys contain yttrium, thorium, copper, nickel, iron, or molybdenum, thereby complicating toxicological attribution. Pulmonary fibrosis, memory and sensory deficits, and increased mortality due to lung cancer have been identified following occupational exposure to dusts generated in the hard metal industry [10]. Historically however, the respiratory and neurological effects observed in workers using tungsten and cobalt hard metal mixtures were thought to be caused by cobalt, not tungsten [10].

Work by Agarwal *et al.* (2011) indicated that tungsten may increase an individual’s risk of CCVD. In the current investigation, we have looked for further evidence of this association, and specifically the association of tungsten exposure with stroke (a cerebral vascular incident characterized by occlusion by an embolus, thrombus, or cerebrovascular hemorrhage or vasospasm, resulting in ischemia of the brain tissues normally perfused by the damaged vessels). Stroke is currently the fourth leading cause of death in the United States [11]. It has a complex multifactorial etiology involving both genetic and environmental risk factors, of which tungsten exposure may be one.

The National Health and Nutrition Examination Survey (NHANES) constitutes a unique dataset with which to investigate the health effects of tungsten. It contains urinary measures of tungsten for 8614 participants aged between 18 and 74 years in six cross sectional data sets spanning twelve years (1999–2009). For the current investigation we have used data from all six waves to investigate the human health effects of tungsten in adults.

## Materials and Methods

### Study Population

NHANES assesses the health and diet of the non-institutionalized US civilian population and is administered by the US National Center for Health Statistics, Centers for Disease Control and Prevention. The study protocol for NHANES was approved by the National Centers for Health Statistics Institutional Review Board and full details of NHANES survey design are available elsewhere [12]. Respondents included in our analyses were those aged 18 to 74 years (of those randomly selected by NHANES for urinary heavy metal measurement) providing a focus on adult health conditions. The 74 year age cut-off was selected to minimize biases caused by non-representation of seniors in institutions. Data were pooled from six independent cross-sectional NHANES waves 1999–2000, 2001–2002, 2003–2004, 2005–2006, 2007–2008 and 2009–2010 which provided a sample of 8,614 adults (18–74 years) for whom urinary tungsten values were available.

### Chemical Analyses

The US National Toxicology Program selected a range of potentially harmful chemicals for human health for further study in specimens from NHANES respondents. Heavy metals, including tungsten were measured in the urine of a subsample of eligible NHANES participants using inductively coupled plasma-mass spectrometry (ICP-MS). This involves the dilution of urine samples (1+9) with 2% (v/v), double-distilled, concentrated nitric acid containing both iridium (Ir) and rhodium (Rh) for multi-internal standardization as originally described by Mulligan *et al* [13]. The same methodology of urinary heavy metal analysis has been employed across all six NHANES waves utilized in this study. A lower limit of tungsten detection was 0.018 ng/ml and within the pooled data set 503 individuals had urinary tungsten concentrations below the lower limit of detection. For analysis the urinary tungsten concentration was first expressed as µg/mg of urinary creatinine and subsequently log transformed.

### Disease Outcomes

Disease outcomes were investigated in NHANES using a questionnaire. Participants were asked “Has a doctor or other health professional ever told you that you have…” for angina, coronary heart disease, heart attack, stroke, asthma, diabetes, emphysema, chronic bronchitis; arthritis, thyroid problems, any kind of liver condition, or cancers. We concentrated particularly on cardiovascular disease (CVD) and stroke, as tungsten has been implicated previously in CCVD. We defined CVD as any reported diagnosis of angina, heart attack or coronary heart disease. We investigated stroke separately from CVD as each constitutes a distinct global health issue. The self-reported diagnoses could not be confirmed via medical records. However, the derived stroke and CVD variables were associated with classical risk factors in our NHANES demographic (Table S1). NHANES also has information on the year of diagnosis for CVD and stroke. For each NHANES wave we therefore calculated the number years since diagnosis of CVD and stroke at the time of recruitment to NHANES.

### Statistical Analysis

Initially we calculated age and sex adjusted weighted means of tungsten concentration for individuals with and without a stroke or CVD diagnosis using a student t-test. We also investigated how the concentration of tungsten varied as time since diagnosis increased. Logistic regression models were then used to estimate odds ratios (OR) and its 95% confidence interval (CI), to characterize a potential association between tungsten exposure (continuous variable) and either stroke or CVD (both binary outcomes). Both crude and multivariable models were fitted, with the latter adjusting for potential confounders (including age, sex, ethnicity, socio-economic status (investigated using education and the poverty income ratio), smoking status (adjusted for both packs per year and serum cotinine), alcohol consumption, occupation, clinical factors (BMI (classified as underweight, normal, overweight, obese and very obese), hypertension and hypercholesterolemia), molybdenum and cobalt concentration.). For this procedure we defined hypertension as either a systolic blood pressure greater than 140 mm Hg, a diastolic pressure of greater than 90 mm Hg or a self-reported prior diagnosis by a physician. Hypercholesterolemia was defined as a blood cholesterol >240 mg/dL or self-reported as a prior diagnosis by a physician.

We further adjusted our models for other diseases reported in NHANES including cardiovascular disease or stroke (depending on our outcome variable), diabetes and emphysema. We also adjusted for a range of other heavy metals including (barium, cadmium, lead, uranium and thallium).

Association between CVD or stroke and tungsten was investigated using logistic regression, in the overall pooled data using NHANES waves 1999–2009. To determine the reproducibility of our findings we also investigate the association between urinary tungsten and stroke or CVD in each NHANES wave. Each wave represents a separate cohort and therefore if association is noted in more than one wave it increases the validity of our findings.

In the pooled data we stratified our analyses by a) age to investigate the association in the under 50 s (18–50 years) and b) sex. We also performed a sensitivity analysis to investigate the associations when the 503 individuals with urinary tungsten measures below the lower detection limit included.

As a sensitivity analysis we adjusted for urinary creatinine levels in our models to allow for dilution effects, and for other variables in the model (e.g., age, sex, race/ethnicity) to be independent of effects of urinary creatinine concentration [14].

All analyses were weighted for the complex cluster sample design and conducted using STATA/IC Version 12.1 (College Station, US). Sampling errors were estimated using the *Taylor Series Linearization* method to account for stratification and clustering.

## Results

**Figure 1:**
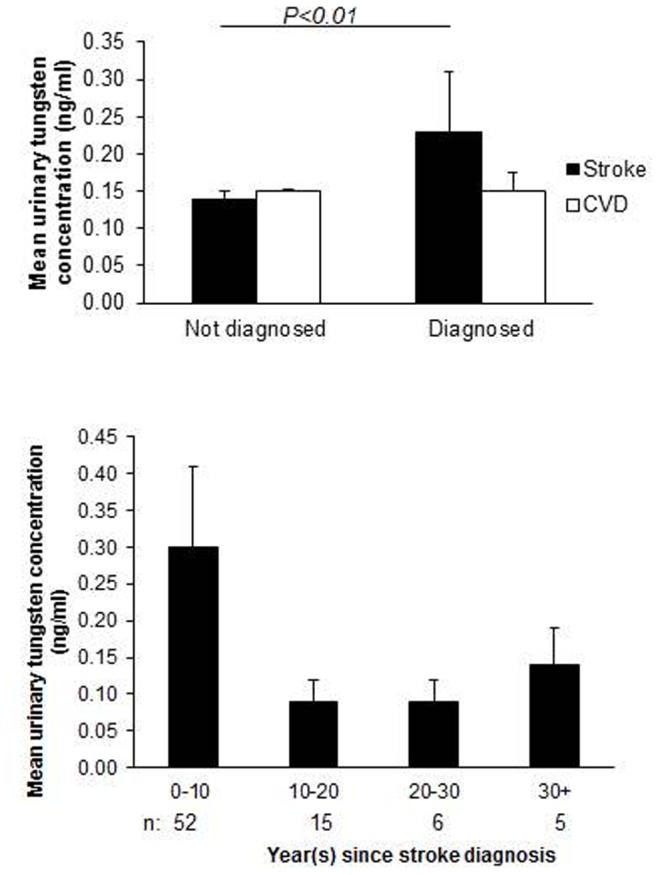
Bar charts representing the mean urinary tungsten concentrations in individuals with and without a self-reported stroke or CVD diagnosis. Error bars represent the 95% confidence intervals and the *P* value represents the comparison of tungsten concentrations in people with and without a stroke diagnosis.

From 1999–2010 there is variation in the urinary tungsten concentration with post 2003/4 waves having higher urinary tungsten levels than other years (Table 1). Urinary tungsten concentrations were altered significantly by race (higher in Non-Hispanic Blacks and Hispanic Mexicans), BMI (highest in those with low BMI) and socioeconomic status (higher in low income, poorer educated individuals). Urinary tungsten concentration was higher in the 203 individuals reporting a stroke diagnosis (0.25 versus 0.13 ng/ml *P* = 0.06; Figure 1). As time since diagnosis increased, the urinary tungsten concentration decreased (*P*<0.05; Figure 1).

**Table 1:** Demographics of the pooled dataset and the weighted mean urinary tungsten concentration for the various demographic variables.

	Pooled data	Tungsten Concentration	Significant variation in Tungsten
		ng/ml (95% CI)	
**N**	8614	0.14 (0.14–0.15)	
**Sex** Male (%)	4223 (49.02)	0.16 (0.14–0.17)	<0.001 (0.278)
Female (%)	4391 (50.98)	0.13 (0.12–0.14)	
**Age (%)**			<0.001 (0.0687)
18–24 years	1682 (19.53)	0.19 (0.16–0.22)	
25–34 years	1530 (17.76)	0.17 (0.14–0.17)	
35–44 years	1512 (17.55)	0.14 (0.12–0.15)	
45–54 years	1427 (16.57)	0.14 (0.12–0.16)	
55–64 years	1292 (15.00)	0.11 (0.10–0.12)	
65–74 years	1171 (13.59)	0.12 (0.10–0.14)	
**Race/Ethnicity (%)**			<0.001 (<0.001)
Mexican American	2054 (23.84)	0.17 (0.15–0.18)	
Other Hispanic	408 (4.74)	0.11 (0.10–0.13)	
Non-Hispanic White	3880 (45.04)	0.14 (0.13–0.15)	
Non-Hispanic Black	1962 (22.78)	0.17 (0.15–0.18)	
Other race	310 (3.60)	0.14 (0.12–0.16)	
**Level of Education**			0.315 (0.555)
Less than high school diploma	2652 (30.82)	0.15 (0.14–0.17)	
High school diploma	2178 (25.31)	0.14 (0.13–0.16)	
Some college education	3775 (43.87)	0.14 (0.13–0.15)	
Unknown	9 (0.10)		
**Annual Income**			0.343 (0.510)
<$20,000	1230 (14.28)	0.16 (0.13–0.18)	
$20,000 to $35,000	1121 (13.01)	0.14 (0.12–0.16)	
$35,000 to $75,000	1719 (19.96)	0.13 (0.11–0.15)	
>$75,000	1099 (12.76)	0.13 (0.11–0.15)	
Unknown	3445 (39.99)	0.16 (0.14–0.17)	
**Body mass index (BMI)**			0.0146 (0.002)
Low weight, BMI<18.5	169 (1.96)	0.18 (0.12–0.23)	
Recommended, BMI 18.5–24.9	2566 (29.79)	0.15 (0.14–0.17)	
Overweight, BMI 25.0–29.9	2872 (33.34)	0.14 (0.12–0.15)	
Obese I, BMI 30.0–34.9	1586 (18.41)	0.13 (0.12–0.14)	
Obese II, BMI> = 35.0	1289 (14.96)	0.15 (0.13–0.16)	
Unknown	132 (1.53)	0.13 (0.08–0.17)	
**Smoking Status (pack year)**			0.411 (0.643)
Never	1971 (22.88)	0.12 (0.11–0.14)	
<20 years	938 (10.89)	0.14 (0.11–0.16)	
20 to 39 years	497 (5.77)	0.12 (0.10–0.13)	
> = 40 years	153 (1.68)	0.11 (0.10–0.15)	
Unknown	5055 (58.68)	0.16 (0.15–0.17)	
**Survey Year**			<0.001 (0.001)
1999–2000	1336 (15.51)	0.14 (0.13–0.16)	
2001–2002	1533 (17.80)	0.13 (0.11–0.16)	
2003–2004	1496 (17.37)	0.12 (0.10–0.13)	
2005–2006	1390 (16.14)	0.16 (0.14–0.17)	
2007–2008	1193 (13.85)	0.16 (0.14–0.17)	
2009–2010	1666 (19.34)	0.15 (0.13–0.17)	

*P* values represent statistical significance of the different tungsten concentrations for each demographic variable. The *P* value presented in () includes an adjustment for urinary creatinine. Statistical comparisons excluded individuals in the unknown categoriesTables.

Our data provided evidence of increased odds of reporting stroke as urinary tungsten concentration in adults (18–74 years) increased (Table 2). Urinary tungsten was associated with stroke in unadjusted (OR: 1.53, 95% CI: 1.14, 2.05) and adjusted models (OR: 1.66, 95% CI: 1.17, 2.34). The inclusion of all urinary tungsten measures did not alter our findings (OR: 1.51, 95% CI: 1.14, 2.00; Table 2). The odds of stroke was not altered when other heavy metals were included in the model (OR: 2.10 (95% CI: 1.24, 3.57)). Stratification by sex in the models demonstrated that tungsten increased an individual’s odds of a stroke in females and significance was approached in males (Male OR: 1.51, 95% CI: 0.92, 2.46; Female OR: 2.07, 95% CI: 1.20, 3.60; Table 2).

**Table 2:** Odds Ratios and 95% confidence intervals representing the odds of a stroke diagnosis per 1 unit increase in log transformed urinary tungsten (expressed as µg per mg of urinary creatinine) for NHANES participants less than 75 years of age or less than 50 years of age.

	Age Range in NHANES Included			
	18–74 years		18–49 years	
Model	Stroke Cases (Controls)	Odds ratio	Stroke Cases (Controls)	Odds ratio
Crude	203 (7,902)	1.53 (1.14–2.05)**	46 (4,801)	1.53 (1.07–2.17)*
Adjusted^a^	64 (3,799)	1.66 (1.17–2.34)**	25 (2,481)	2.17 (1.33–3.53)**
Adjusted^b^	69 (4,084)	1.51 (1.14–2.00)**	28 (2,634)	1.75 (1.26–2.45)**
Males only	35 (2,079)	1.51 (0.92–2.46)^?^	11 (1,304)	Insufficient cases
Females only	29 (1,720)	2.07 (1.20–3.60)*	14 (1,177)	Insufficient cases

a The adjusted models include age, sex, ethnicity, SES, smoking, occupation, BMI, hypertenstion, hypercholesterolemia, molybdenum and cobalt concentration as covariates.

b In this model all urinary tungsten measures were included, including the 503 individuals with a concentration below the lowest detectable limit.

Statistical significance is denoted by ^?^, * and ** representing *P*<0.1, *P*<0.05 and *P*<0.01 respectively.

Stratification by age, revealed that strong associations remained between stroke and urinary tungsten in the under fifties (Table 2; Adjusted OR 2.17, 1.33, 3.53) and these associations remained when other diseases were considered in the model (OR: 4.13 (2.07, 8.24)).

Our findings were not altered when we adjusted for creatinine in the model as supposed to adjusting the urinary tungsten concentration (Table S2).

When we investigated the individual NHANES waves associations were evident between stroke and tungsten in four of the six waves (2001–2002, 2005–2006, 2007–2008 and 2009–2010) in crude models and two of the six (2007–2008 and 2009–2010) in adjusted models (Table S3).

The evidence for association between tungsten and CVD was considerably weaker. Tungsten concentrations were similar in individuals reporting a diagnosis of CVD and individuals without the condition (Figure 1). In the pooled data an association between tungsten and CVD was noted in crude models but not in adjusted models (Table 3). In the individual NHANES waves no association was noted between urinary tungsten and CVD (Table S3).

**Table 3:** Odds Ratios and 95% confidence intervals representing the odds of a CVD diagnosis per 1 unit increase in log transformed urinary tungsten (expressed as µg per mg of urinary creatinine) for NHANES participants less than 75 years of age or less than 50 years of age.

	Age Range in NHANES Included			
	18–74 years		18–50 years	
Model	CVD Cases (Controls)	Odds ratio	CVD Cases (Controls)	Odds ratio
Crude	445 (7,648)	1.17 (1.02–1.34)*	63 (4,779)	0.94 (0.67–1.31)
Adjusted^a^	145 (3,711)	1.11 (0.86–1.43)	28 (2,474)	0.92 (0.56–1.52)
Adjusted^b^	157 (3,989)	1.16 (0.93–1.47)	31 (2,627)	0.99 (0.64–1.55)
Males only	114 (2,121)	1.09 (0.80–1.49)	16 (1,297)	Insufficient cases
Females only	43 (1,868)	1.16 (0.76–1.77)	12 (1,177)	Insufficient cases

a The adjusted models include age, sex, ethnicity, SES, smoking, alcohol consumption, occupation, BMI, hypertenstion, hypercholesterolemia, molybdenum and cobalt concentration as covariates.

b In this model all urinary tungsten measures were included, including the 503 individuals with a concentration below the lowest detectable limit.

Statistical significance is denoted by * representing *P*<0.05.

## Discussion

This study revealed a strong association between urinary tungsten concentrations and stroke. The association between higher tungsten concentrations, which reflect higher environmental and dietary exposures to tungsten, and stroke were consistent and independent of traditional risk factors.

To date, few studies have considered the adverse human health impacts of tungsten or the demographic variables that increase an individual’s risk of elevated tungsten exposure. Using the NHANES dataset we have demonstrated that urinary tungsten concentration varied with both race and BMI. Individuals reporting a stroke diagnosis were noted to have higher levels of urinary tungsten and this appeared to be higher in individuals whose stroke had occurred within the last decade.

### Stroke

Our findings are consistent with an earlier study in which a statistically significant relationship was noted between urinary tungsten and CCVD in NHANES [9]. Our research builds on this earlier work by examining the role of tungsten in both CVD and stroke separately and in a much larger sample. The association between tungsten and stroke was strong, with no attenuation of the OR after adjustment for classical stroke risk factors. The relationship remained when other co-morbidities (CVD, diabetes and emphysema) were included in the model, suggesting that tungsten may be involved in the etiology of stroke in some individuals.

Stratification by age revealed that the association was also evident in individuals less than fifty years old. Stroke predominates in the elderly (those over 65 years of age) while stroke in younger individuals is believed to involve genetic risk factors and have a different etiology (with cardiac embolism predominating over atherosclerosis) [15]. This may indicate that tungsten increases an individual’s risk of stroke through pathway(s) that increase the risk of atherosclerosis and embolism, although it was not possible to investigate the different subtypes of stroke within the NHANES data.

Stratification by gender suggested that tungsten increased the odds of stroke in both males and females, but the OR was higher in females. Whilst men are more likely to have a stroke, more women actually die from strokes [16]. The reason behind this disparity is unknown and cannot be fully explored in this cross sectional study. In this study we had an even distribution of strokes in males and females perhaps suggesting some bias in our data.

Previous studies have indicated that metal toxicity is involved in stroke etiology [17]. The OR for stroke was not attenuated following adjustment for cobalt (often considered to be the principle toxic agent) and molybdenum. Other metals not measured in NHANES, which can be alloyed with tungsten (e.g. yttrium, thorium, copper, nickel) may contribute to the metal toxicity observed and need to be considered further in future investigations. Whilst we cannot rule out their involvement, our findings point to tungsten as being of critical importance in stroke etiology of many of the cases considered here. If not, then tungsten is apparently strongly correlated with some unidentified agent, which itself underpins the development of stroke. Despite the limited power of statistical analysis mentioned earlier tungsten was still associated with stroke in multiple individual NHANES waves. Each wave is a separate cross-sectional cohort and therefore observing the association in more than one wave strengthens the validity of our findings.

We anticipate that tungsten will interfere with biochemical pathways as it can replace molybdenum (Mo) at metal binding sites in proteins [18]. Mo occurs in a wide range of metalloenzymes and in eukaryotes, the most prominent Mo-enzymes are nitrate reductase, sulfite oxidase, xanthine dehydrogenase, aldehyde oxidase, and the mitochondrial amidoximereductase [19]. If tungsten were to replace Mo at the binding site of these proteins, rendering them non- or partially functional, it is biologically plausible that it could increase the risk of disease. For example, the nitrate to nitrite ratio (which is controlled by nitrate oxidase) has been implicated in stroke [20] and is known to be associated with oxidative cellular damage via the production of reactive oxygen species (ROS).

Furthermore tungsten and it’s alloys generate ROS [21] and deplete glutathione in tissues [22], both of which are implicated in stroke [23], [24]. ROS can influence numerous cellular and vascular pathways which are implicated in stroke pathophysiology.

### Cardiovascular Disease

Logistic regression models revealed crude associations between tungsten and CVD, but these were attenuated following adjustment for potential confounders. This suggests that tungsten is not involved in the CVD etiology.

### Limitations

The trends observed could arise due to confounding factors we did not recognize or measured. We did investigate associations in individual waves of NHANES, demonstrating that stroke was associated with urinary tungsten concentration in two waves. The power of this analysis was limited by low numbers of stroke cases reported in this study. Due to the cross-sectional nature of NHANES, we do not have access to data on tungsten levels prior to the development of a specific medical condition. Only single measurements of heavy metal levels were determined, which may not represent chronic exposure. However, urinary heavy metal levels are usually considered as biomarkers of choice when assessing chronic exposure. This study may also be subject to survival bias wherein participants with the diseases of interest or severe risk factors may be less likely to, or unable to participate in the study. In the case of mortality from stroke in young people this would only increase the strength of the association. The exclusion of institutionalized individuals tends to exclude those with severe illnesses following heavy metal exposure. However, this bias is likely to result in an underestimation of the strength of associations between tungsten and stroke.

### Conclusions

Statistically significant correlations were observed between stroke and urinary tungsten level. Independent replication in a prospective cohort is now needed to confirm the associations reported. If the associations reported here are confirmed in independent studies, it should give impetus to work to identify the mechanisms of action linking long-term, low-dose tungsten exposure to adverse outcomes in humans. Given the substantial negative effects on adult health that may be associated with elevated tungsten and also given the potential for reducing human exposure, our findings merit further research into the human health effects of this metal.

## Supporting Information

Table S1 Investigating the association between classical a) stroke risk factors and a self-reported doctor diagnosis of stroke and b) classical CVD risk factors and a self-reported doctor diagnosis of CVD in the six pooled NHANES waves for all adults aged 18–74 with valid urinary tungsten measures.
(DOCX)

Table S2 **Odds Ratios and 95% confidence intervals representing the odds of a stroke diagnosis per 1 unit increase in urinary tungsten adjusting for creatinine in the model for NHANES participants less than 75 years of age or less than 50 years of age.**
(DOCX)

Table S3 **Odds ratios and 95% confidence intervals representing the odds of a stroke or CVD diagnosis per 1 unit increase in log transformed urinary tungsten concentration (expressed as µg per mg of urinary creatinine) for each of the six NHANES waves in adults aged less than 75 years.**
(DOCX)
