# Supplementary material for: High Urinary Tungsten Concentration Is Associated with Stroke in the National Health and Nutrition Examination Survey 1999–2010
Source: PLoS One. 2013 Nov 11;8(11):e77546. doi: 10.1371/journal.pone.0077546 (PMC3823878; doi:10.1371/journal.pone.0077546)
Supplement: Table S1 — Investigating the association between classical a) stroke risk factors and a self-reported doctor diagnosis of stroke and b) classical CVD risk factors and a self-reported doctor diagnosis of CVD in the six pooled NHANES waves for all adults aged 18–74 with valid urinary tungsten measures. (DOCX) [file pone.0077546.s001.docx]

| **Table S1a** |  | |  |  |
| --- | --- | --- | --- | --- |
| **Risk Factor** | **Odds ratio for stroke (95% confidence intervals)** | | **P** |  |
| Age | 1.07 (1.06-1.08) | | **<0.001** |  |
| Sex (risk related to female gender) | 1.34 (0.96-1.88) | | 0.085 |  |
| Hypertension | 1.41 (1.02-1.98) | | **0.041** |  |
| Diabetes | 5.76 (3.79-8.76) | | **<0.001** |  |
| Hypercholesterolemia | 1.65 (1.05-2.60) | | **0.031** |  |
| **Table S1b** |  |  | | |
| **Risk Factor** | **Odds ratio for CVD (95% confidence intervals)** | **P** | | |
| Age | 1.09 (1.08-1.10) | **<0.001** | | |
| Sex (risk related to female gender) | 0.69 (0.53-0.92) | **0.010** | | |
| Hypertension | 1.41 (1.05-1.89) | **0.021** | | |
| Diabetes | 5.83 (4.41-7.70) | **<0.001** | | |
| Body mass index | 1.04 (1.03-1.06) | **<0.001** | | |

Table S1. Investigating the association between classical a) stroke risk factors and a self-reported doctor diagnosis of stroke and b) classical CVD risk factors and a self-reported doctor diagnosis of CVD in the six pooled NHANES waves for all adults aged 18-74 with valid urinary tungsten measures.
