# Supplementary material for: High Urinary Tungsten Concentration Is Associated with Stroke in the National Health and Nutrition Examination Survey 1999–2010
Source: PLoS One. 2013 Nov 11;8(11):e77546. doi: 10.1371/journal.pone.0077546 (PMC3823878; doi:10.1371/journal.pone.0077546)
Supplement: Table S2 — Odds Ratios and 95% confidence intervals representing the odds of a stroke diagnosis per 1 unit increase in urinary tungsten adjusting for creatinine in the model for NHANES participants less than 75 years of age or less than 50 years of age. (DOCX) [file pone.0077546.s002.docx]

|  | **Age Range in NHANES Included** | | | |
| --- | --- | --- | --- | --- |
|  | **18-74 years** | | **18-49 years** | |
| **Model** | **Stroke Cases**  **(Controls)** | **Odds ratio** | **Stroke Cases**  **(Controls)** | **Odds ratio** |
| Crude | 203  (7,902) | 1.57 (1.17-2.12)** | 46  (4,801) | 1.95 (1.32-2.88)** |
| Adjusted^a^ | 64  (3,799) | 1.95 (1.45-2.63)*** | 25  (2,481) | 2.43 (1.70-3.48)*** |
| Adjusted^b^ | 69  (4,084) | 1.94 (1.44-2.62)*** | 28  (2,634) | 2.42 (1.69-3.47)*** |

Table S2: Odds Ratios and 95% confidence intervals representing the odds of a stroke diagnosis per 1 unit increase in urinary tungsten adjusting for creatinine in the model for NHANES participants less than 75 years of age or less than 50 years of age.

^a^The adjusted models include age, sex, ethnicity, SES, smoking, occupation, BMI, hypertenstion, hypercholesterolemia, molybdenum and cobalt concentration as covariates. ^b^ In this model all urinary tungsten measures were included, including the 503 individuals with a concentration below the lowest detectable limit. Statistical significance is denoted by *, ** and ** representing *P*<0.05, *P*<0.01 and *P*<0.001 respectively.
