# Supplementary material for: High Urinary Tungsten Concentration Is Associated with Stroke in the National Health and Nutrition Examination Survey 1999–2010
Source: PLoS One. 2013 Nov 11;8(11):e77546. doi: 10.1371/journal.pone.0077546 (PMC3823878; doi:10.1371/journal.pone.0077546)
Supplement: Table S3 — Odds ratios and 95% confidence intervals representing the odds of a stroke or CVD diagnosis per 1 unit increase in log transformed urinary tungsten concentration (expressed as µg per mg of urinary creatinine) for each of the six NHANES waves in adults aged less than 75 years. (DOCX) [file pone.0077546.s003.docx]

Table S3.Odds ratios and 95% confidence intervals representing the odds of a stroke or CVD diagnosis per 1 unit increase in log transformed urinary tungsten concentration (expressed as µg per mg of urinary creatinine) for each of the six NHANES waves in adults aged less than 75 years.

|  | **NHANES Year** | | | | | |
| --- | --- | --- | --- | --- | --- | --- |
|  | **1999/2000** | **2001/2002** | **2003/4** | **2005/6** | **2007/8** | **2009/10** |
|  | **Stroke** | | | | | |
| Odds ratio (95% CI) | 0.72 (0.45,1.16) | 1.63 (1.00-2.67)? | 1.30 (0.87-1.93) | 1.51 (1.04-2.21)* | 1.52 (1.13-2.05)** | 1.54 (0.97-2.46)? |
| Stroke cases crude | 31 (1152) | 22 (1332) | 41 (1263) | 29 (1172) | 43 (1442) | 37 (1541) |
| Adjusted odds ratio (95% CI) | 0.59 (0.24,1.45) | 1.61 (0.75-3.45) | 1.24 (0.69-2.24) | 1.57 (0.88-2.80) | 1.74 (1.10-2.77)* | 1.84 (1.01-3.34)* |
| Stroke cases adjusted | 22 (918) | 17 (1167) | 36 (1137) | 25 (1065) | 34 (1237) | 33 (1316) |
|  | **Cardiovascular disease** | | | | | |
| Odds ratio (95% CI) | 1.51 (1.00-2.29)? | 1.14 (0.89-1.47) | 1.20 (0.89-1.61) | 1.04 (0.73-1.48) | 1.15 (0.86-1.53) | 1.07 (0.71-1.62) |
| CVD cases crude | 77 (1104) | 78 (1275) | 90 (1214) | 59 (1331) | 79 (1402) | 67 (1599) |
| Adjusted odds ratio (95% CI) | 1.50 (0.91-2.47) | 1.29 (0.95-1.76) | 1.07 (0.70-1.65) | 1.10 (0.74-1.65) | 1.15 (0.70-1.88) | 1.08 (0.64-1.82) |
| CVD cases adjusted | 61 (878) | 66 (1118) | 78 (1095) | 51 (1194) | 62 (1205) | 58 (1362) |

Statistical significance is denoted by ?, * and ** representing *P*<0.1, *P*<0.05 and *P*<0.01 respectively. The adjusted models include age, sex, ethnicity, SES, smoking, alcohol consumption, occupation, BMI, hypertenstion, hypercholesterolemia, molybdenum and cobalt concentration as covariates.
